# Ultrasound-Assisted Salt Penetration in Sauced Duck: Insights from LF-NMR and MRI Combined Analysis

**DOI:** 10.3390/foods14203553

**Published:** 2025-10-18

**Authors:** Xiangyu Wang, Chenlan Xia, Huimin Li, Yangying Sun, Daodong Pan, Jun He

**Affiliations:** College of Food Science and Engineering, Ningbo University, Ningbo 315832, China; 18858947067@163.com (X.W.); xiachenlan2004@outlook.com (C.X.); li15716331226@163.com (H.L.); sunyangying@nbu.edu.cn (Y.S.)

**Keywords:** ultrasonic, salt distribution, curing, marinating, meat processing

## Abstract

This study investigated the effect of ultrasound-assisted curing on salt penetration in sauced duck using a combination of low-field nuclear magnetic resonance (LF-NMR) and magnetic resonance imaging (MRI). Ultrasound treatment significantly accelerated salt penetration in duck meat during curing. The salt content of ultrasound-treated samples at 150 W, 300 W, and 450 W was 2.56 ± 0.08%, 2.84 ± 0.02%, and 3.52 ± 0.02%, respectively, significantly higher than that of the untreated control 2.17 ± 0.09%. Moreover, the enhancing effect on salt uptake increased with ultrasound power. Notably, treatment with 28 kHz, 450 W ultrasound resulted in a salinity comparable to that achieved by traditional curing 3.46 ± 0.11%. To further assess salt distribution, LF-NMR and MRI were employed, providing non-destructive, rapid, and precise visualization of salt penetration. Pseudo-color images confirmed the salt content results and revealed that the 28 kHz, 450 W ultrasound treatment promoted a more uniform salt distribution, similar to conventionally marinated samples. These findings indicate that the combined use of LF-NMR and MRI is a promising approach for characterizing and monitoring salt penetration in duck meat. Overall, this study provides valuable insights for improving and controlling the quality of highly processed meat products.

## 1. Introduction

Duck meat products—such as sauced duck, salt-cured duck, roast duck, and smoked duck—are highly valued by consumers, particularly in Asian countries, for their rich nutritional profile and distinctive flavor [1]. Salting is a critical step in the production of these products, during which duck meat is either marinated in a salt solution or dry-cured with stir-fried salt, allowing salt ions to diffuse into the muscle [2]. This process plays a key role in shaping both the flavor and overall quality of duck meat. However, conventional salting methods face persistent challenges, including slow penetration, uneven distribution, and excessive salt uptake, in addition to being time-consuming. Therefore, developing more efficient salting techniques holds significant potential for advancing the duck meat industry.

Ultrasound technology, as a novel, green, and environmentally friendly processing method, has been increasingly applied in the food industry [1]. In recent years, it has been widely explored in meat processing and quality control [3]. The primary mechanism underlying its effects is acoustic cavitation, in which microbubbles form and collapse during ultrasonication. This phenomenon generates localized high temperatures and pressures, thereby enhancing mass transfer between the sample and its solvent and ultimately influencing salt uptake [4,5]. For example, low-intensity ultrasound (0–1 W/cm^2^, >100 kHz) is a non-destructive technique that can be used to monitor changes in the physical and chemical properties of foods during processing [6,7,8,9]. For example, Gómez-Salazar et al. [10] reported that low-intensity ultrasound (40 kHz, 110 W) accelerated the salting process in fresh rabbit meat, increasing its salt content while reducing the negative effect of salt concentration on the a*-value (redness value). Since ultrasound treatment often decreases a* values, which can negatively affect meat color, this finding is particularly relevant. Moreover, ultrasound has also been investigated for marinating low-sodium beef [11] and ham [12], further demonstrating its potential to enhance salt diffusion and improve curing efficiency. Duck meat is relatively common in marinated products, and its muscle fibers may respond differently to ultrasonic treatment compared with pork, beef, or lamb. Nevertheless, limited research has focused on the effect of low-intensity ultrasound on salt penetration in duck meat.

As emerging analytical technologies, low-field nuclear magnetic resonance (LF-NMR) and magnetic resonance imaging (MRI) have been increasingly applied in food research, particularly for the characterization of water, fat, and protein [13]. LF-NMR typically employs compact permanent magnets and operates at frequencies below 60 MHz [14]. It detects the radiofrequency absorption of spin-½ nuclei (mainly protons) in an external static magnetic field, and correlates spin–lattice (T_1_) and spin–spin (T_2_) relaxation times with proton distribution and mobility in food matrices, thereby enabling the evaluation of water content and related physicochemical properties. In contrast, MRI is a non-invasive, non-ionizing imaging technique that reconstructs spatial images from the interactions between atomic nuclei and magnetic fields [13]. Together, LF-NMR and MRI provide powerful tools for visualizing water distribution and molecular states in food systems. For example, LF-NMR and MRI have been used to assess water distribution in pork meatballs under different treatments [15] and in chicken breast [16]. Because salt diffusion in muscle may be related to water migration [17], these techniques are potentially useful for investigating salt penetration. However, limited research has applied these techniques to duck meat. To bridge this gap, the present study utilized LF-NMR and MRI to examine the effects of ultrasound on salt penetration in braised duck.

Previous studies have shown that ultrasound can enhance mass transfer. We hypothesized that ultrasound treatment could promote salt penetration in sauced duck and that there is a correlation between the distributions of moisture and salt. Therefore, the present study aimed to investigate the effects of ultrasound on salt penetration and to propose a visualization method of salt distribution using a combination of LF-NMR and MRI. The findings are expected to provide valuable insights for evaluating salting efficiency and optimizing duck meat processing.

## 2. Materials and Methods

### 2.1. Preparation of Sauced Ducks

**Figure 1:**
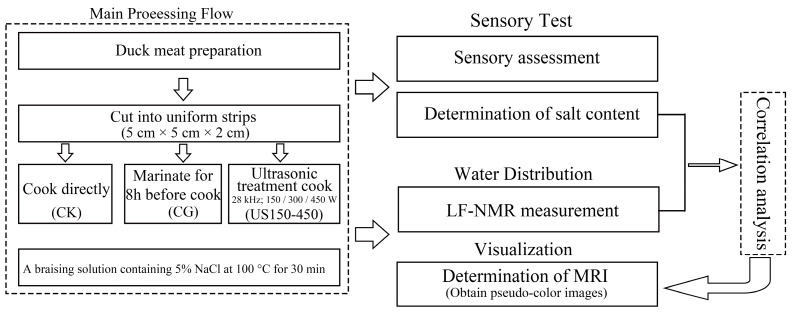
Schematic representation of the experimental procedure.

The experimental procedure followed the flowchart shown in Figure 1. A total of 30 Fresh Cherry Valley ducks (100–105 days old, 1.5–1.8 kg) were obtained from a local market (Ningbo, Zhejiang, China). Duck breasts were trimmed to remove peripheral connective tissue and visible fat, cut into uniform strips (5 cm × 5 cm × 2 cm), and then randomly allocated to the respective groups, with each group consisting of 8 replicates. The brine solution (per 10 L of water) consisted of 80 g glutamate, 80 g sugar, 50 g soy sauce, 40 mL cooking wine, 40 mL White wine, 20 g clove, 20 g zoonosis pilosula root, 15 g lemongrass, 15 g fennel, 15 g cinnamon, 15 g ginger, 5 g chili, 5 g woody fragrance, 4 g hawthorn, 4 g nutmeg, 2 g villous amomum fruit, 2 g gardenia, and 2 g tsaoko. The pre-cut duck meat strips were divided into five treatment groups. In the control group (CK), samples were cooked directly in a braising solution containing 5% NaCl at 100 °C for 30 min without prior marination. In the curing group (CG), samples were immersed in the same 5% NaCl braising solution and marinated for 8 h, followed by cooking at 100 °C for 30 min. For the ultrasound-assisted groups, samples were cooked in a 5% NaCl braising solution at 100 °C for 30 min in an ultrasonic bath operating at 28 kHz, with power levels of 150 W (US150), 300 W (US300), and 450 W (US450), respectively. The corresponding ultrasound intensities were 0.33 W/cm^2^, 0.67 W/cm^2^, and 1.0 W/cm^2^, respectively. All duck breast samples were randomly allocated to the respective groups, with each group including 8 replicates. Cooked samples, except those used for sensory evaluation, were stored at −40 °C until further analysis.

### 2.2. Sensory Assessment

The sensory evaluation followed the methodology of Cho et al. [18], and was adapted to create a quantitative descriptive analysis (QDA) table (Table S1). After training with triangle test, ten trained panelists (5 males and 5 females, aged 25–35 years) then independently evaluated the sensory attributes of each sample, including saltiness, aroma, tenderness, color, and juiciness. Each attribute was rated on a 9-point intensity scale, where 1 represented the lowest and 9 the highest intensity. To ensure objectivity and accuracy, panelists were instructed to cleanse their palates between samples.

### 2.3. Determination of Salt Content

The salt content of the sauced duck was determined using a modified method based on Contreras-Lopez et al. [19]. In brief, 10.0 g of meat sample was homogenized with 90.0 mL of distilled water using a high-speed homogenizer (DY 89-I, Xinzhi Biotechnology Co., Ltd., Ningbo, China) at 4 °C and 32,000× *g* for 30 s. The resulting homogenate was allowed to stand at room temperature for 30 min and then filtered. The salt concentration of the filtrate was measured using a digital salinometer (AR8012, Xima Instruments Co., Ltd., Dongguan, China) with a detection range of 0% to 10% and an accuracy of 0.001%. The salt content of the sauced duck was calculated based on these measurements.

### 2.4. Determination of LF-NMR

LF-NMR relaxation measurements were performed according to a previously reported method [20], using a 20.76 MHz LF-NMR analyzer (NMI20-040H-I, Niumag Analytical Instrument Co., Ltd., Suzhou, China). Briefly, 2.0 g of each sample was placed in a cylindrical glass tube (15 mm diameter, 15 mL capacity) and inserted into the NMR probe. Transverse relaxation (T2) measurements were conducted using the Carr-Purcell-Meiboom-Gill (CPMG) pulse sequence at an operating temperature of 32 ± 0.01 °C [21,22]. The main operating conditions were as follows: time waiting = 4000 ms, time echo = 0.1 ms, number of echoes = 8000, number of scans = 8, spectral width = 200 kHz, sweep frequency = 20 MHz, pulse 1 width = 7 µs, pulse 2 width = 14 µs. And the following parameters were used for processing: filter level = 1, inversion points = 200, relaxation time points = 100, Tmin (ms) = 0.01, Tmax (ms) = 10,000, and iterations = 100,000. The CPMG decay curves were fitted and subjected to simultaneous iterative reconstruction technique (SIRT) inversion calculation. After mass normalization, T_2_ spectra (T_21_, T_22_, and T_23_) and the corresponding water populations were obtained. Each sample was measured three times, and each group was measured in triplicate.

### 2.5. Determination of MRI

Non-destructive MRI analysis was performed to obtain proton density-weighted images of the samples using a spin-echo (SE) sequence. With slight modifications based on Yang et al. [23], duck meat samples were wrapped in raw rubber tape and placed in a 15 mm diameter glass tube for imaging. Images were acquired and analyzed using Image Evaluation software (version 3.0, NMI20-040H-I, Niumag Analytical Instrument Co., Ltd., Suzhou, China). The imaging parameters were as follows: echo time (TE) = 10.46 ms, repetition time (TR) = 1000 ms, slice thickness = 8 mm, and slice width = 3 mm to ensure comprehensive scanning of the samples. The resonance frequency was set at 20.76 MHz, the magnetic field strength at 0.5 T, and the temperature was maintained at 32 ± 0.01 °C.

### 2.6. Statistical Analysis

Experimental data were analyzed using one-way ANOVA followed by Duncan’s multiple range test with SAS software (Version 8.0, SAS Institute Inc., Cary, NC, USA). Results are presented as mean ± standard deviation, and graphs were generated using Origin 2017 (OriginLab, Northampton, MA, USA). Statistical significance was defined at *p* < 0.05.

## 3. Results and Discussion

### 3.1. Sensory Attributes

**Figure 2:**
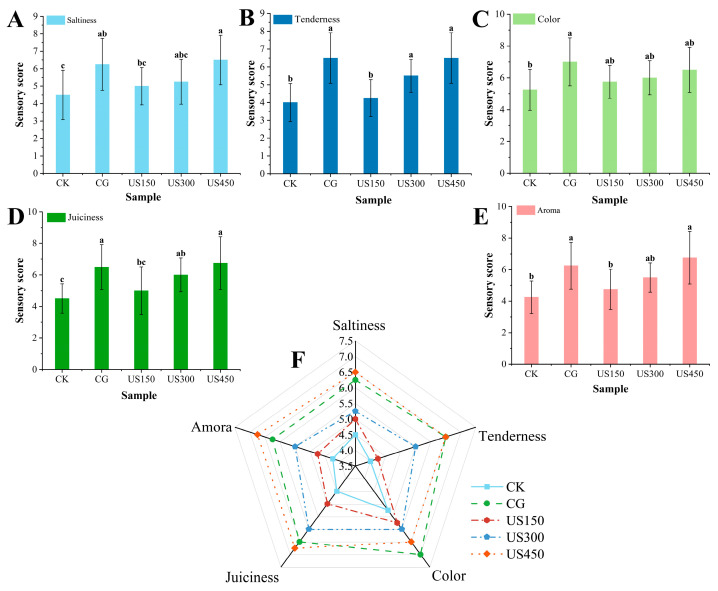
Sensory evaluation scores of sauced ducks under different ultrasound treatments. CK: control group; CG: curing group; US150, US300, and US450: ultrasound-assisted curing at 150, 300, and 450 W, respectively. Attributes assessed: saltiness (**A**), tenderness (**B**), color (**C**), juiciness (**D**), and aroma (**E**). A radar chart comparing overall sensory attributes is shown in (**F**). Different letters indicate significant differences among treatments (*p* < 0.05).

Salt and saltiness play a critical role in the quality and flavor of most foods, particularly meat products. Previous studies have reported that ultrasound can accelerate salt penetration in pork [2]. In the present study, ultrasound treatments at varying powers were applied to investigate their effects on salt diffusion and distribution in sauced duck. As shown in Figure 2A, high-power ultrasound (US450, 6.50 ± 1.41) significantly enhanced the saltiness of sauced duck (*p* < 0.05) and exhibited a similar effect on salt diffusion, compared with low-power treatments (US150 and US300, 5.00 ± 1.06 and 5.25 ± 1.28, respectively) and the control (CK, 4.50 ± 1.41). This finding aligns with Ojha et al. [24], who reported that ultrasound enhances salt diffusion in meat muscle compared with static brining in pork processing. Similar effects have also been observed in chicken [25]. The ultrasound mechanism is most pronounced at the liquid–solid interface, where it can alter muscle structure and potentially improve tenderness [26,27,28]. In our study, sensory evaluation (Figure 2B) indicated slight improvements in perceived tenderness; however, these observations are based on sensory assessment and supporting literature. As shown in Figure 2C,E, ultrasound treatment had no significant effect on flavor or color [29], although variations in juiciness were observed across different ultrasound powers (Figure 2D). Overall changes in the meat samples from all treatments are summarized in a radar chart (Figure 2F), highlighting the significant impact of ultrasound on sauced duck.

**Figure 3:**
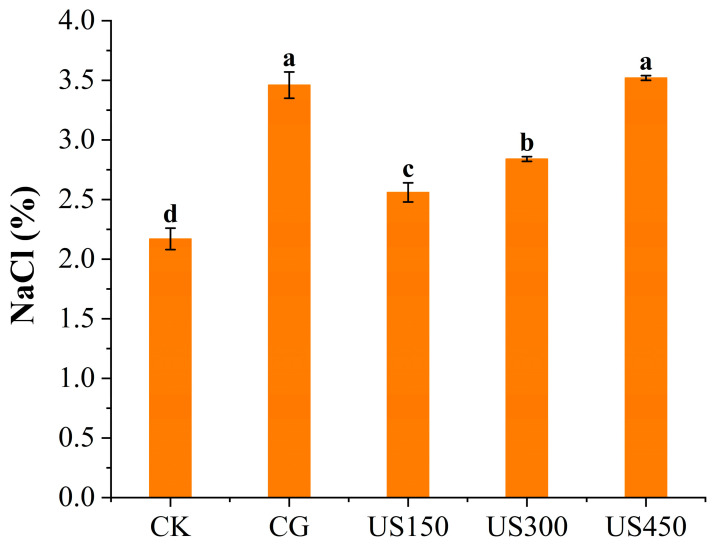
Effect of different processing methods on the salt (NaCl) content of sauced duck. CK: control group; CG: curing group; US150, US300, and US450: ultrasound-assisted curing at 150, 300, and 450 W, respectively. Different letters denote significant differences among treatments (*p* < 0.05).

To confirm the accelerating effect of ultrasound on salt diffusion, the NaCl content of each sauced duck sample was measured and is shown in Figure 3. Consistent with the sensory evaluation results, US450 (3.52 ± 0.02%) exhibited a NaCl content similar to that of the CG (3.46 ± 0.11%), which was significantly higher than those of the other treatments (*p* < 0.05). The enhanced NaCl transfer induced by ultrasound is likely attributable to microjets that accelerate NaCl diffusion in muscle, expel air through cavitation, and enlarge voids within muscle fibers, thereby increasing salt content [30]. A similar study investigated the effects of ultrasound-assisted curing on salt distribution in lamb under different ultrasound intensities (0, 19.55, 24.57, and 27.54 W·cm^−2^), revealing that treatments at 24.57 and 27.54 W·cm^−2^ significantly enhanced salt content compared to static curing [31]. In addition, the actual salt content of US300 (2.84 ± 0.02%) was significantly higher than that of US150 (2.56 ± 0.08%, *p* < 0.05). However, no significant difference in perceived saltiness was observed between US150 and US300 in the sensory evaluation (*p* > 0.05), which may be attributed to the limited resolution of human sensory perception. These findings indicate that ultrasound-assisted treatment is a promising method for enhancing salt penetration in sauced duck. However, further studies are warranted to evaluate the uniformity of salt distribution within the meat.

### 3.2. Food Compositional Analysis by LF-NMR

#### 3.2.1. Distribution of Water

**Figure 4:**
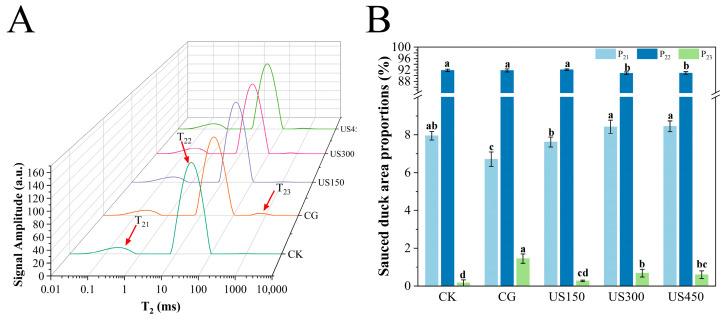
Water distribution in sauced duck under different ultrasonic treatments, analyzed by LF-NMR. (**A**) T_2_ relaxation spectra showing signal intensities of distinct water populations (T_21_, T_22_, and T_23_). (**B**) Relative proportions of the water populations (P_21_, P_22_, and P_23_). CK: control group; CG: curing group; US150, US300, and US450: ultrasound-assisted curing at 150, 300, and 450 W, respectively. Different letters indicate significant differences among treatments (*p* < 0.05).

LF-NMR technology is commonly used to provide information on proton distribution and dynamics in samples [32]. In this study, three peaks were identified in each sauced duck meat sample under different treatments using LF-NMR T_2_ distribution fitting curves to assess hydrogen proton relaxation times (Figure 4) [33]. The relaxation components were assigned as follows: T_21_ (0.01–10 ms), representing bound water closely associated with macromolecules; T_22_ (10–100 ms), representing immobilized water trapped within the myofibrillar cytosolic network with limited mobility; and T_23_ (100–1000 ms), representing free water located in the extracellular space, indicates the increased mobility of free water molecules [34]. In general, shorter T_2_ relaxation times reflect stronger interactions between water and macromolecules.

Figure 4A shows the T_2_ relaxation time distribution of the samples under different treatments, reflecting changes in internal water status. Compared with the CK, the T_23_ relaxation times of the other groups were prolonged, indicating changes in free water [35]. Regarding water distribution, the proportions of bound water (P_21_) and free water (P_23_) in the CG were lower and higher, respectively, than those in the control and the three ultrasound groups (*p* < 0.05). Among the ultrasound treatments, US300 and US450 exhibited similar effects on water dynamics, which was also reflected in the immobilized water data: the P_22_ ratios of US300 and US450 were significantly lower than those of the other groups (*p* < 0.05), highlighting the pronounced effect of ultrasound at specific intensities on water distribution in sauced duck meat (Figure 4B). The NaCl in the marinade enhances water fluidity, while ultrasound treatment induces structural changes in the meat, facilitating deeper NaCl penetration [36]. This phenomenon is primarily attributed to cavitation during ultrasound processing. Gas release and disruption of muscle tissue create larger gaps between muscle bundles, promoting brine penetration. Additionally, negative pressure within the muscle reduces resistance to marinade absorption, allowing it to diffuse more effectively [37]. As a result, water and proteins maintain more uniform contact, indirectly increasing water fluidity [38]. These findings support the use of moisture-related data to further investigate salt distribution in sauced duck samples.

#### 3.2.2. Correlation Analysis

Pearson’s correlation analysis was performed to explore the relationships between LF-NMR parameters and salt content in sauced duck meat. The results revealed moderate negative correlations between the relaxation times T_21_, T_22_, and T_23_ and salt content, with correlation coefficients of −0.580, −0.574, and −0.552, respectively (*p* < 0.05). No significant correlations were observed between salt content and the proportions of bound water (P_21_) or immobilized water (P_22_) (*p* > 0.05). In contrast, the proportion of free water (P_23_) exhibited a strong positive correlation with salinity (r = 0.843, *p* < 0.01), and total water content (P_total_) showed a strong negative correlation with saltiness (r = −0.822, *p* < 0.01). These relationships can be attributed to the osmotic effects of salt, as water and salt are closely interdependent within muscle tissue. Changes in salt content significantly affect water retention and overall muscle quality. Previous studies have demonstrated that increased salt concentrations alter the osmotic pressure of muscle fibers, leading to continuous water loss and subsequently affecting textural and functional properties [39,40]. Collectively, these findings highlight the significant interplay between water and salt in sauced duck meat, providing a basis for the subsequent visualization analyses.

### 3.3. Visualization of Salt Diffusion

**Figure 5:**
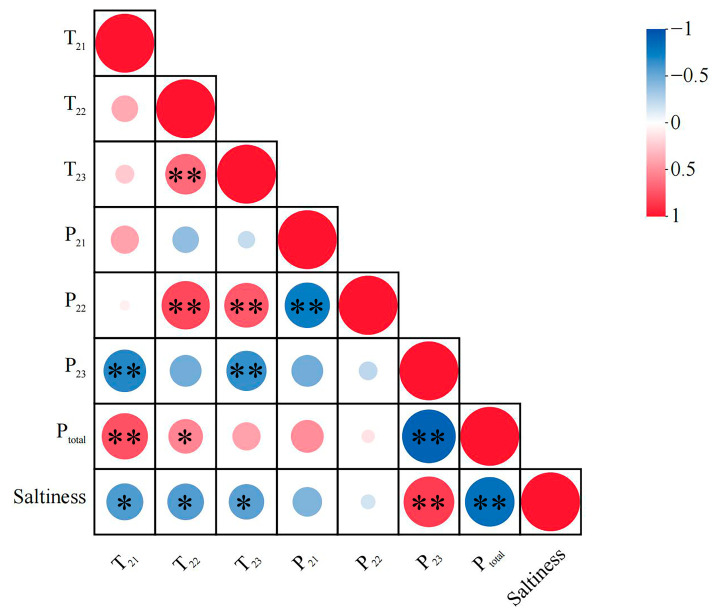
Pearson correlation analysis between water relaxation parameters, water populations, total water content, and saltiness in sauced duck. The size and color of the circles indicate the strength and direction of the correlations (red: positive; blue: negative). Asterisks (*) denote correlation coefficients, with significance levels marked as * *p* ≤ 0.05, ** *p* ≤ 0.01.

**Figure 6:**
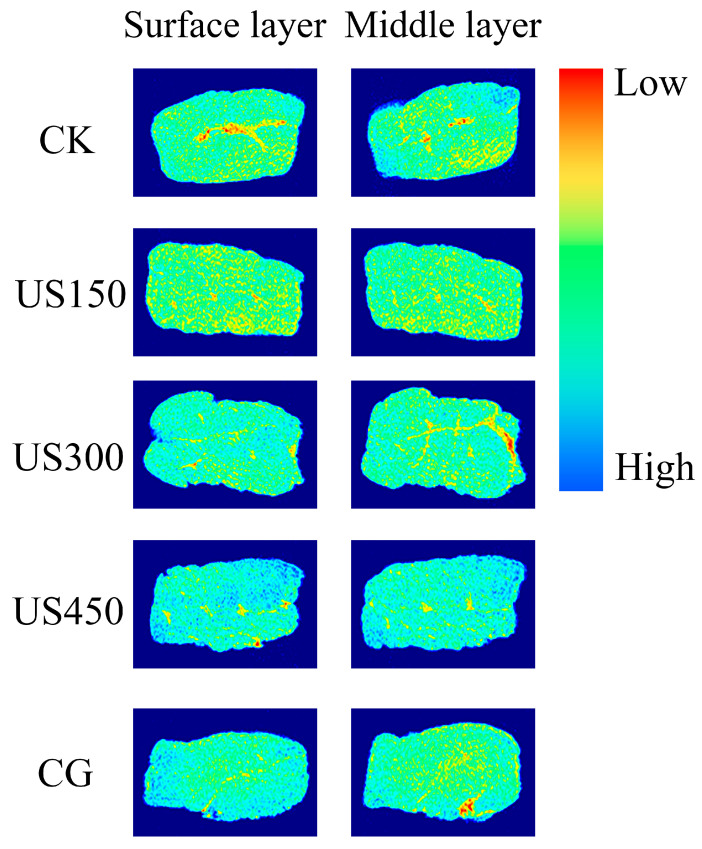
Proton density-weighted MRI analysis of salt distribution in duck breast under different ultrasound treatments. CK: control group; CG: curing group; US150, US300, and US450: ultrasound-assisted curing at 150 W, 300 W, and 450 W, respectively. The color scale on the right represents proton intensity, where red indicates high signal strength (low salt content) and blue indicates low signal strength (high salt content).

MRI is a powerful tool for visually investigating the spatial distribution of molecules within food matrices [41], enabling non-destructive monitoring of relevant molecular content. The combination of LF-NMR (T_2_) and MRI has been widely applied to analyze and visualize water status and distribution in meat products [42,43]. Moreover, the strong correlation between salt and water allows the assessment of salt diffusion in meat using this approach [31,44]. In the present study, a strong negative correlation was observed between salt content and T_2_ (r = −0.822, *p* < 0.01) in sauced duck (Figure 5). Consequently, MRI was employed to visually examine salt diffusion in duck meat subjected to ultrasound treatments. Figure 6 presents pseudo-color images of the sauced duck samples under different treatments, where regions of varying colors (red, yellow, green, and blue) correspond to proton density and water content from high to low, which inversely reflects salt density. These images generally agree with the salt content measurements, indicating that ultrasound promotes salt penetration, with group CG and US450 exhibiting higher salinities than the other groups (CK, US150 and US300). This consistency suggests that the combination of LF-NMR and MRI is a feasible approach for investigating and visualizing salt distribution in duck meat. Furthermore, both superficial and internal sections were analyzed to characterize salt distribution within the samples. As expected, pseudo-color images show lower salt content in the central regions compared with the edges (Figure 6), further supporting the utility of this two-technology approach for monitoring internal salt distribution and homogeneity. Notably, the images also reveal more uniform salt diffusion in US450 than in CG, despite comparable overall salinities. This suggests that ultrasound may be more effective than traditional marination in achieving salt homogenization, which may be related to protein degradation and denaturation during ultrasound treatment, facilitating salt penetration [41,45]. Similar observations were reported by Manzocco et al. [46] who estimated salt content during ham curing using MR signal values, confirming the potential of MRI for predicting salt content in meat products. The MRI provides non-destructive internal imaging and, with further technical optimization and cost reduction, its application in the food industry is expected to expand significantly in the near future.

## 4. Conclusions

This study demonstrated that ultrasound treatment significantly enhanced salt penetration and distribution in sauced duck, with the best performance achieved at 28 kHz and a power of 450 W. Compared with CK, CG, US150, and US300, US450 markedly improved salt penetration efficiency. Notably, although CG and US450 exhibited similar salt contents, US450 achieved this result in a shorter time, highlighting its potential advantage in terms of improving processing efficiency. Moreover, LF-NMR and MRI enabled rapid, non-destructive evaluation of salt content and diffusion efficiency in the samples. The results confirm that ultrasound effectively promotes salt diffusion and absorption while mitigating uneven penetration in meat products. These findings highlight the potential of ultrasound-assisted curing to improve salt distribution and penetration in processed meats. However, several limitations should be noted, including the relatively small sample size, the lack of texture and microbiological analyses, and the inherent limitations of sensory evaluation. Future studies should address these aspects and incorporate more detailed flavor assessments to comprehensively evaluate the effects of ultrasound treatment on overall product quality, thereby providing stronger support for its wider application in the meat processing industry.

## Data Availability

The data presented in this article are available from the corresponding authors upon request. The data are not publicly available due to privacy and ethical restrictions.

## Supplementary Materials

The following supporting information can be downloaded at: https://www.mdpi.com/article/10.3390/foods14203553/s1, Table S1. Quantitative descriptive analysis of sensory attributes of sauced duck.
