# Supplementary material for: Ultrasound-Assisted Salt Penetration in Sauced Duck: Insights from LF-NMR and MRI Combined Analysis
Source: Foods. 2025 Oct 18;14(20):3553. doi: 10.3390/foods14203553 (PMC12564898; doi:10.3390/foods14203553)
Supplement: Supplementary file 1 [file foods-14-03553-s001.zip › Supplementary Materials.pdf]

Supplementary Materials

Table S1 Quantitative descriptive analysis of sensory attributes in sauced duck

Attribute	Description	Standard	Scoring Details (1-9)
Saltiness	Intensity of saltiness	Moderate, well-distributed saltiness; overly salty or bland samples scored lower	1 = very poor, 3 = poor, 5 = fair, 7 = good, and 9 = excellent
Tenderness	Softness and chewability	Tender texture with good chewiness; no toughness	
Color	Uniformity and visual appeal of meat color	Uniform reddish-brown appearance with surface gloss	
Juiciness	Juiciness perceived during chewing	Moderate juiciness and moist mouthfeel	
Aroma	Aroma of meat and seasoning	Strong braised aroma with a distinct natural duck flavor; no off-flavor	
